# Predicting long-term sickness absence among retail workers after four days of sick-listing

**DOI:** 10.5271/sjweh.4041

**Published:** 2022-10-01

**Authors:** Corné AM Roelen, Erwin EM Speklé, Birgit I Lissenberg-Witte, Martijn W Heymans, Willem van Rhenen, Frederieke G Schaafsma

**Affiliations:** 1 Arbo Unie, Occupational Health Service, Utrecht, The Netherlands; 2 University Medical Center Groningen, University of Groningen, Department of Health Sciences, Groningen, The Netherlands; 3 Amsterdam University Medical Centers, VU University Amsterdam, Department of Public and Occupational Health, Amsterdam Public Health Research institute, Amsterdam, The Netherlands; 4 Amsterdam University Medical Centers, VU University Amsterdam, Department of Epidemiology and Data Science, Amsterdam, The Netherlands; 5 Nyenrode Business University, Center for Leadership and Management Development, Breukelen, The Netherlands

**Keywords:** external validation, occupational health service, prediction model, risk assessment, ROC analysis, sick leave

## Abstract

**Objective:**

This study tested and validated an existing tool for its ability to predict the risk of long-term (ie, ≥6 weeks) sickness absence (LTSA) after four days of sick-listing.

**Methods:**

A 9-item tool is completed online on the fourth day of sick-listing. The tool was tested in a sample (N=13 597) of food retail workers who reported sick between March and May 2017. It was validated in a new sample (N=104 698) of workers (83% retail) who reported sick between January 2020 and April 2021. LTSA risk predictions were calibrated with the Hosmer-Lemeshow (H-L) test; non-significant H-L P-values indicated adequate calibration. Discrimination between workers with and without LTSA was investigated with the area (AUC) under the receiver operating characteristic (ROC) curve.

**Results:**

The data of 2203 (16%) workers in the test sample and 14 226 (13%) workers in the validation sample was available for analysis. In the test sample, the tool together with age and sex predicted LTSA (H-L test P=0.59) and discriminated between workers with and without LTSA [AUC 0.85, 95% confidence interval (CI) 0.83–0.87]. In the validation sample, LTSA risk predictions were adequate (H-L test P=0.13) and discrimination was excellent (AUC 0.91, 95% CI 0.90–0.92). The ROC curve had an optimal cut-off at a predicted 36% LTSA risk, with sensitivity 0.85 and specificity 0.83.

**Conclusion:**

The existing 9-item tool can be used to invite sick-listed retail workers with a ≥36% LTSA risk for expedited consultations. Further studies are needed to determine LTSA cut-off risks for other economic sectors.

Long-term sickness absence (LTSA) is a major problem with high societal costs in Organization for Economic Cooperation and Development (OECD) countries (1). LTSA is associated with financial and psychological problems among workers and may lead to future unemployment and societal exclusion (2). The LTSA risk increases with sickness absence duration and, therefore, workers at risk of LTSA are preferably identified before they report sick. However, prediction models for future LTSA in non-sick-listed workers show poor-to-moderate results (3–6), probably because of the low LTSA prevalence in the general working population. The performance is not sufficient to identify and target workers at risk of LTSA for preventive actions (7).

LTSA is more prevalent and may be better predicted among workers who report sick. Recently, Louwerse et al (8) developed an LTSA prediction model for sick-listed individuals without an employment contract. In The Netherlands, unemployed individuals report sick to UWV, the Dutch social security agency. A prediction model including educational level, expected sickness absence duration and problems (yes/no) with seeking help, distinguished between individuals with and without sickness absence one year later. The area under the curve (AUC) was 0.76 which indicates that in all random pairs of individuals, the prediction model correctly identified the one at highest LTSA risk in 76% of the cases. The authors concluded that the prediction model can be used to identify unemployed individuals with an increased risk of LTSA after reporting sick.

A prediction model for unemployed individuals, however, may not apply to employed workers. Dutch workers with an employment contract report sick to their employer, who asks an occupational health service (OHS) to advise about work accommodations and return-to-work (RTW) activities. For this purpose, sick-listed workers are usually invited to an OHS consultation in the fourth or fifth week of sickness absence. Arbo Unie is an OHS that provides occupational healthcare to 1.2 million workers employed in companies of different economic sectors throughout The Netherlands. Arbo Unie sends a brief (9-item) online tool to sick-listed workers four days after reporting sick to collect information about symptoms and reasons for sickness absence.

The objective of the present study was to test and validate the existing 9-item online tool for its ability to identify workers with an increased LTSA risk after four days of sick-listing.

## Methods

### Study context and design

In The Netherlands, employers financially compensate sickness absence for a maximum period of two years. Workers report sick to their employer, who records the first day of sickness absence into an OHS sickness absence register. Sickness absence dates are not registered real time, usually there is a delay of one or more days if employers do not register sickness absence in the OHS database on a daily basis.

The present study analyzed data which were collected within the context of usual occupational healthcare practice. The online tool has been in use for several years in the retail sector. A convenience sample (N=13 597) of food retail workers who reported sick between March and May 2017 was used to test the online tool for its LTSA risk predictions. LTSA risk predictions were validated in a sample of 104 698 workers who reported sick between January 2020 and April 2021. They were employed in retail (83%), transport (6%), healthcare (3%), industry (2%) and various other economic sectors (6%). The medical ethics committee of the Amsterdam University Medical Centers granted ethical clearance for the study.

### Predictor variables

The 9-item tool is completed online by the sick-listed worker on the fourth day of sickness absence.

The tool asked the worker about expectations of sickness absence duration (<1 week=1, 1–2 weeks=2, 2–5 weeks=3, >5 weeks=4), contact with the supervisor (no=0, yes but no RTW arrangements made=1, and yes and RTW arrangements made=2), sickness absence in the past 12 months (no=0, yes once=1, yes more than once=2), fatigue in the past 4 weeks (5-point frequency scale ranging from never=0 to always=4), pain in the past 4 weeks (5-point frequency scale ranging from never=0 to always=4), physical work demands (5-point frequency scale ranging from not at all=1 to very high=5), mental work demands (5-point frequency scale ranging from not at all=1 to very high=5), opportunities to accommodate work (no=0, yes=1), and whether or not a medical doctor (general practitioner or clinician) was visited (no=0, yes=1 but no treatment; and yes, receive treatment=2).

Age and sex were retrieved from the OHS sickness absence register and used as additional predictor variables.

### Outcome variable

The day of reporting sick and the day of full RTW were retrieved at the individual level from the OHS sickness absence register. The duration of sickness absence was used to determine the outcome variable. Dutch sickness absence policies require the employer to contact the OHS if sickness absence is expected to exceed a duration of 6 weeks. In line with these policies, LTSA was defined in this study as sickness absence ≥6 weeks. Thus LTSA=0 for sickness absence durations <6 weeks and LTSA=1 for durations ≥6 weeks.

### Statistical analysis

All statistical analyses were conducted in SPSS Statistics for Windows, version 26.0 (IBM Corporation, Armonk, NY, USA). The online tool could only be returned when all items were completed. Consequently, there were no missing responses on the tool’s items.

In the test sample, age, sex and the 9 items of the online tool were included as independent variables in a multivariable logistic regression model with LTSA as outcome variable. The constant and regression coefficients of this multivariable logistic regression model were used to compute a linear predictor (LP) variable in the validation sample. LP was used as a single independent variable in logistic regression analysis of LTSA in the validation sample (9).

Calibration (ie, the accuracy of LTSA predictions) was investigated in both (test and validation) samples with calibration curves and the Hosmer-Lemeshow (H-L) goodness-of-fit test. A non-significant H-L P-value reflects that the predicted risks did not deviate significantly from the observed frequencies, indicating that calibration is adequate (9). Discrimination (ie, the ability to distinguish between workers with and without LTSA) was investigated in both samples with receiver operating characteristic (ROC) analysis, regarding the area under the ROC curve (AUC) as measure for discrimination (9). Perkins & Schisterman (10) stated that the “optimal” cut-off point in ROC analyses is the point which classifies most of the individuals correctly. The ‘index of union’ method proposed by Unal (11) was used to determine the optimal cut-off point.

We conducted a subgroup analysis investigating calibration and discrimination in the 17% non-retail workers of the validation sample to get an idea about the tool’s performance in economic sectors other than retail.

## Results

**Figure 1:**
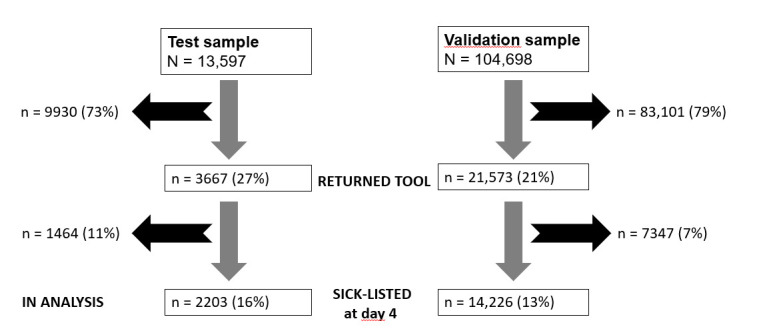
Recruitment of test and validation samples.

In the test sample, 9930 (73%) workers did not return the online tool; the OHS sickness absence register showed that most of them had resumed work within days and maximum one week of reporting sick. Of the 3667 workers who returned the tool, 1464 workers reported that they had already resumed work and were therefore excluded from the analyses. The data of the 2203 (16%) workers who were still sick-listed four days after reporting sick was used for testing LTSA risk predictions and discrimination by the 9-item online tool (figure 1). In the validation sample, 83 101 (79%) workers did not return the online tool. Of the 21 573 workers who returned the tool, 7347 reported they had resumed work. The data of 14 226 (13%) workers who were still sick-listed four days after reporting sick was used for validating LTSA risk predictions and discrimination by the existing 9-item online tool (figure 1).

Table 1 shows the study population characteristics and the online tool scores in the test and validation samples. In the validation sample, fewer workers expected to be absent for ≥5 weeks and more workers had had contact with their supervisor. Fatigue, physical work demands, and mental work demands were lower in the validation compared to the test sample.

**Table 1:** Study population characteristics. [LTSA=long-term sickness absenc; SD=standard deviation]

	Test sample								Validation sample							
	
	Total (N=2203)				LTSA (N=875)				Total (N=14 226)				LTSA (N=2156)			
			
	Mean	SD	N	%	Mean	SD	N	%	Mean	SD	N	%	Mean	SD	N	%
Age	33.3	15.4			34.4	15.0			33.6	16.0			40.4	15.9		
Sex																
Men			831	38			325	37			5150	36			724	34
Women			1372	62			550	63			9076	64			1432	66
Expected sickness absence duration (weeks)																
<1			758	34			94	11			4322	30			97	4
1–2			511	23			196	22			5673	40			321	15
3–5			465	21			235	27			2714	19			817	38
>5			469	21			350	40			1517	11			921	43
Contact with supervisor																
No			231	11			72	8			96	1			13	1
Yes, but no return to work arrangements			890	40			499	57			6938	49			1509	70
Yes, and return to work arrangements			1082	49			303	35			7192	50			634	29
Prior sickness absence ^a^																
No			589	39			350	40			5070	36			735	34
Yes, once			894	41			338	39			5892	41			866	41
Yes, more than once			450	20			187	21			3264	23			555	25
Fatigue (0–4) ^b^	1.9	1.3			2.1	1.3			1.7	1.2			2.2	1.3		
Pain (0–4) ^b^	1.5	1.3			1.9	1.4			0.9	1.2			1.6	1.4		
Physically demanding work (1–5) ^c^	2.1	1.1			2.3	1.2			1.0	1.0			1.6	1.3		
Mentally demanding work (1–5) ^c^	1.8	1,1			2.1	1.3			0.6	0.9			1.1	1.3		
Work accommodations																
No			1656	75	683	78					10 285	72			1618	75
Yes			547	25	192	22					3941	28			538	25
Medical doctor visit																
No			349	16	68	8					6945	49			468	22
Yes, but no treatment			1339	61	499	57					4773	34			815	38
Yes and treatment			515	23	308	35					2508	18			873	40

a In the past 12 months.

b 0 = never and 4 = always.

c 1 = not at all and 5 = very much.

### Testing LTSA risk predictions and discrimination (N=2203)

In the test sample, 875 (40%) workers had LTSA. In multivariable analysis, the worker-reported expectation of sickness absence duration had the highest Wald statistic, indicating that it explains the highest part of the variance in LTSA (table 2). Older age, contact with supervisor without RTW arrangements, pain, mentally demanding work, and medical treatment were associated with a significantly higher LTSA risk. Sex, fatigue, prior sickness absence, physically demanding work, and opportunities to accommodate work were not significantly associated with the risk of LTSA.

**Table 2:** Multivariable logistic regression analysis in the test sample. (N=2203) [OR=odds ratio; CI=confidence interval].

	Wald ^a^	OR	95% CI
Age	7.72	1.01	1.00–1.02
Sex	0.20		
Men		1	
Women		1.05	0.85–1.33
Expected sickness absence duration (weeks)	390.16		
<1		1	
1–2		3.73	2.68–5.20
3–5		10.35	7.41–14.45
>5		34.35	23.50–50.21
Contact with supervisor	23.05		
No		1	
Yes, but no return to work arrangements		1.54	1.04–2.27
Yes, and return to work arrangements		0.87	0.59–1.29
Prior sickness absence	0.90		
No		1	
Yes, once		0.89	0.70–1.14
Yes, more than once		0.97	0.72–1.31
Fatigue	0.30	1.03	0.93–1.14
Pain	5.65	1.12	1.02–1.23
Physically demanding work	0.06	0.99	0.87–1.11
Mentally demanding work	5.73	1.17	1.02–1.32
Work accommodations	1.31		
No		1	
Yes		0.86	0.67–1.11
Medical doctor visit	5.29		
No		1	
Yes, but no treatment		1.22	0.85–1.74
Yes and treatment		1.55	1.03–2.31

a Wald statistic=(regression coefficient / standard error)^2^

**Figure 2:**
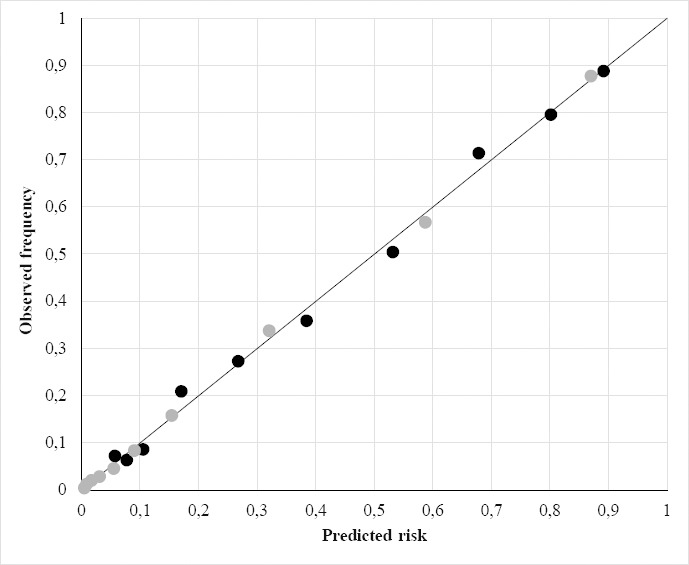
Calibration plot. The figure plots the predicted long-term sickness absence (LTSA) risk against the observed LTSA frequency in deciles of the development sample (black dots, N=2203) and the validation sample (grey dots, N=14 226); the diagonal represents perfect calibration.

The H-L test (model χ^2^=6.53; df=8; P=0.59) reflected adequate calibration (figure 2). Discrimination between retail workers with and without LTSA was good, with an AUC 0.85 [95% confidence interval (CI) 0.83–0.87]. If the worker-reported expectation of sickness absence duration was eliminated from the model, the AUC decreased to 0.75 (95% CI 0.73–0.77). If contact with the supervisor was eliminated from the model, the AUC remained 0.85 (95% CI 0.83–0.86). The interaction terms ‘age*physically demanding work’, ‘age*mentally demanding work’, ‘sex*physically demanding work’ and ‘sex*mentally demanding work’ did not improve discrimination [data not shown].

### Validating LTSA risk predictions and discrimination (N=14 226)

In the validation sample, 2156 (15%) workers had LTSA. The median predicted LTSA risk was 7.0% (interquartile range 1.6–31.6%). The H-L test was not significant (model χ^2^=12.41; df=8; P=0.13), ie, the calibration of LTSA risk predictions was adequate (figure 2). Discrimination was excellent with AUC 0.91 (95% CI 0.90–0.92). The ‘index of union’ method showed that the optimal cut-off point was at a 36% LTSA risk, with sensitivity 0.85 and specificity 0.83.

In the subgroup of 4092 (17%) non-retail workers, there were 1130 LTSA events. The H-L test was χ^2^=8.42 (P=0.39) and the AUC was 0.89 (95% CI 0.88–0.91). The optimal cut-off point was at a 29% LTSA risk, with sensitivity 0.84 and specificity 0.79.

## Discussion

The existing 9-item online tool together with age and sex predicted the risk of LTSA after four days of sick-listing and discriminated well between sick-listed retail workers with and without LTSA. In a validation sample, LTSA risk predictions by the tool were adequate and discrimination was excellent.

The results of this study confirmed that worker-reported expectation of sickness absence duration is a strong LTSA predictor (8, 12, 13). If the worker-reported expectation of sickness absence duration was eliminated from the analysis, discrimination diminished from excellent to moderate. Thus, we need this variable to identify workers with an increased LTSA risk. Contact with supervisor was a second strong predictor variable that was positively related to LTSA when no RTW arrangements were made, and negatively though not significantly when there were RTW arrangements. Previously, Buys et al (14) reported that supervisor contacts facilitate RTW if sick-listed workers perceive the contacts as supportive. The lower LTSA risk found in the present study may be explained by supportive arrangements, for example, work accommodation strategies to make RTW easier. Supervisor contacts without RTW arrangements were associated with a significantly higher LTSA risk. It is well conceivable that agreeing on RTW arrangements is difficult if supervisors and workers have no idea about when RTW will be possible. If supervisor contact was eliminated from the analyses, discrimination did not change, indicating that the predictor variable was not essential for discriminating between retail workers with and without LTSA.

The other predictor variables may also be not essential for identifying workers at risk of LTSA four days after sick-listing. Nevertheless, we decided against removing predictor variables from the tool because the objective of the study was to test the tool as it is used now in occupational healthcare practice, not to develop a parsimonious LTSA risk prediction model. In line with the systematic review by Dekkers-Sanchez et al (15), age but not sex was associated with LTSA. Prior sickness absence was associated with a higher risk of LTSA in univariable analysis [data not shown], but lost significance in the multivariable model. The same holds for fatigue and physically demanding work. Mentally demanding work and pain were significantly associated with a higher LTSA risk. Higher LTSA risks were also found for medical doctor visits. A recent systematic review and meta-analysis of the literature showed that workers on paid sick leave have higher odds of seeing a doctor (16). Workers who receive treatment or are referred for clinical diagnostics and treatment are likely to have more severe disorders and thus a higher LTSA risk than those who don’t need any treatment.

In prediction research, the overall performance of the multivariable model is more important than the association of individual predictor variables with the outcome (9). Calibration and discrimination are the most commonly used model performance indicators. Previous studies have investigated LTSA risk predictions in the healthy working population. Prediction models for risk of LTSA among non-sick-listed workers show adequate calibration but poor-to-moderate discrimination with AUCs varying between 0.68 (4) and 0.76 (6). Among sick-listed unemployed workers, Louwerse et al (8) reported discrimination by a three-predictor (educational level, expected sickness absence duration, and help-seeking ability) model, with an over-optimism adjusted AUC of 0.76. The present study confirms that workers with an increased risk of LTSA can be identified early after sick-listing. Instead of adjusting for over-optimism, we evaluated LTSA risk predictions in a new validation sample. Although not as good as in the test sample, the tool’s predictions were still adequate in the validation sample. Discrimination between workers with and without LTSA in the validation sample was better than in the test sample.

### Limitations of the study

The study had a two-sample design. The test sample (N=13 597) was a convenience sample of workers in food retail who reported sick between March and May 2017. The validation sample (N=104 698) included workers (83% retail) who reported sick between January 2020 and April 2021. Consequently, the tool’s validation was temporal (ie, testing the validity of the tool over time) rather than external (ie, testing the tool in a different working population). Subgroup analysis showed that the tool also discriminated between non-retail workers with and without LTSA, though the LTSA cut-off risk was lower than in retail workers. This indicates that the tool could work in other economic sectors, but further validation studies are needed to determine LTSA cut-off risks for inviting workers to expedited consultations with occupational healthcare providers.

The response rate was low, 27% and 21% in the test and validation samples, respectively. Consequently, there is a drastic selection process in terms of who is left for testing and validating the tool. Sickness absence register analyses showed that most non-responders in the test sample had already resumed work. There was a 40% LTSA prevalence in the test sample as compared with a 10.7% LTSA prevalence in the Dutch workforce in 2018 (17). If workers with LTSA are overrepresented, the regression coefficients used for the LP variable might overestimate the LTSA risk in new populations with lower LTSA prevalences. Indeed, we found overestimations of the LTSA risk in the validation sample with a 15% LTSA prevalence, but overall the predicted LTSA risks did not deviate significantly from the observed LTSA frequencies. Further research is needed to investigate if LTSA risks are adequately predicted in more heterogeneous samples with workers employed in more diverse economic sectors. Compared to calibration, discrimination between sick-listed workers with and without LTSA will be less affected by non-response bias. ROC curves are independent of the prevalence of the outcome, because the curves are based on sensitivity (true-positive rates among those with the outcome) and specificity (true-negative rates among those without the outcome).

In contrast to the test sample, we had no knowledge about the workers in the validation sample who did not complete the online tool. In line with the test sample, it is likely that most workers in the validation sample did not respond because they had already resumed work or were thinking about resuming work. Alternatively, individuals with severe disease may want to focus on recovery rather than work. Some, particularly older individuals might have difficulties completing an online tool because they don’t have a computer or internet. On the one hand, low response rates implicate a high risk of selection bias, which restricts the reproducibility and generalizability of the results. On the other hand, response rates were low in both samples and might be inherent to occupational healthcare practice. Response rates in primary and occupational healthcare are known to be low and declining (18, 19). By testing and validating the tool in the selective group of responders, we have insight in the tool’s predictive performance in occupational healthcare practice as it is now. Response rates could be improved, for example by other ways of data collection such as telephone interviews. In that case, however, structural changes are needed in the organization of OHS healthcare services.

### Practical implications

A 9-item online tool predicted the risk of LTSA among sick-listed workers after four days of sick-listing. In the present study, we found an optimal cut-off (ie, minimum misclassification) at a 36% and 29% LTSA risk among retail and non-retail workers, respectively. Sick-listed workers with a predicted LTSA risk above the cut-off should be invited to expedited OHS consultations to further explore the reasons for sickness absence and RTW barriers. A recent systematic review showed promising results for interventions addressing barriers and RTW facilitators (20). Occupational healthcare providers could play an important role in a participatory approach involving the sick-listed worker and the supervisor to solve RTW barriers early after sick-listing, thus expediting RTW and potentially preventing LTSA (21).

At a 36% LTSA risk, sensitivity was 0.85 for retail workers, which means that 15% of those with LTSA were missed for expedited consultations. They were invited for an OHS consultation as usual in the fourth or fifth week of sickness absence. If it is important not to miss workers at high risk of LTSA, then occupational healthcare providers could choose a lower LTSA risk with higher sensitivity as cut-off point. They should bear in mind, however, that the number of false positives increases with lower LTSA risk cut-off points. Consequently, more workers are invited for consultations, while they will not develop LTSA. This might result in medicalization of transient symptoms and unnecessary utilization of OHS healthcare services. Therefore, we recommend to carefully consider the pros and cons of LTSA cut-off risks for practical purposes.
